# Report on Tumors of the Jaws and Their Operative Treatment

**Published:** 1860-01

**Authors:** 


### ARTICLE XVI.

### *Report on Tumors of the Jaws and their Operative Treatment.*

[From Medical Times and Gazette, Sept. 3, 1859.]

During the last six years numerous cases have been mentioned in our Hospital Reports (statistical and otherwise) illustrating the pathology and surgery of tumors of the upper and lower jaw. These we now propose to bring together in a concise summary. We will take first those cases requiring severe surgical operations; and these it will be convenient to divide into two classes; first, those requiring resection of the upper jaw; and, second, those in which large portions of the lower jaw were removed. In both of these we of course leave unnoticed cases of necrosis, as the operations required in them are trivial as compared with those in which morbid growths are present. A third series of operative cases will comprise cases of epulis, and after this we shall, under their different heads, briefly review such other affections as occasionally require surgical interference.

Resection of the upper maxilla is emphatically one of the triumphs of modern surgery. It is but thirty years since its first performance, and during that space of time it has been instrumental in saving many lives. Its victims have besides been very few, experience having proved that it is not really the very formidable procedure which conjecture might suppose. To Mr. Lizars (1827) of Edinburgh, is due the credit of having been the first to suggest and attempt it; and to Mr. Syme (1829) that of having been the first British surgeon who performed it. In France, however, it would appear to have originated simultaneously, without any knowledge on the part of its proposer, of Mr. Lizars' case. In the same year as the latter was attempted, M. Gensoul, of Lyons, performed it, we believe, successfully. Since then it has been repeated by many surgeons; amongst whom we must mention prominently Mr. Syme, Mr. Fergusson, and Mr. O'Shaugnessy. The latter surgeon, in Indian practice, successfully employed it for the extirpation of an unusually large mass of morbid growth.

The following is a seriatim list of such cases as have been reported in our pages during the last six years:—

RESECTIONS OF THE UPPER JAW.

Case 1.—St. Bartholomew's Hospital, July, 1853.—In this case, Mr. Lawrence excised the left upper maxilla, on account of malignant disease, commencing in the antrum. The patient recovered well, as far as the operation was concerned, but the disease rapidly returned.

Case 2.—St. Bartholomew's Hospital, October, 1853.—Mr. Stanley extirpated the entire bone with the exception of its nasal and orbital processes on account of a fibroid tumor which had returned after a former excision. The patient recovered well, and the extirpation was, we believe, permanently successful.

Case 3.—Charing-cross Hospital, October, 1853.—Mr. Hancock. In this instance the excision was a complete one, the disease being suspectedly of a malignant nature. The patient recovered well.

Case 4.—Guy's Hospital, November, 1853.—A man aged 50, under the care of Mr. Cock, on account of malignant disease, involving the antrum, and upper maxilla. Almost the entire bone was removed, and with a good result, inasmuch as the man quickly recovered. We believe, however, that the disease recurred within a short period.

Case 5.—St. Thomas' Hospital.—A married woman, aged 53, was admitted into St. Thomas' Hospital on November 29, 1853, under the care of Mr. Le Gros Clark. Her disease was a large malignant growth involving right upper jaw. The growth had been excised by a provincial surgeon about five months before, but had rapidly recurred. The whole duration of the disease was two years and a half. Her general health was still pretty good. Mr. Clark performed excision of the affected maxilla, only the floor of the orbit being left. There was but little hemorrhage. The woman made an uninterruptedly good recovery. She was still quite well when she came under notice again, six months afterwards, but we believe that eventually the diseasc returned.

Case 6.—The Derby Infirmary, 1854.—The patient was a man aged 49, in tolerably good general health. He was under the care of Mr. Fox, of the Derby Infirmary, for a tumor which had distended the antrum, and which had existed for nearly a year. The tumor was believed to be malignant, and it had grown forwards so as to protrude the cheek, and backwards so as to depress the soft palate. The case had been roughly treated by a quack doctor, prior to the man's admission at the hospital, who had made incisions into it from the mouth, and had applied caustics. As no fungoid growths had followed these measures, it was hoped that the disease was not of the most malignant type. Mr. Fox excised the entire maxilla, adopting the plan of operating advised by Mr. Fergusson. After removal of the bone, such portions of the growth as had passed upwards or backwards were carefully removed by the scoop. The characteristics of medullary cancer were well marked in the tumor. The incisions in the skin soon healed, and the chasm left was rapidly filled by granulations, which were, however, of somewhat suspicious character. At the time the man left the hospital there was reason to fear that the the disease was already returning.

Case 7.—King's College Hospital, 1856.—A girl aged 13, in good health, was admitted under Mr. Fergusson's care, on account of an osseous growth distending the left antrum. It had been observed to be increasing for three years. Excision of the greater part of the left upper maxilla was performed, the cheek having been dissected up after a single incision through the upper lip. In the osseous mass was one of the incisor teeth. The girl recovered well, and with very little resultant deformity.

Case 8.—King's College Hospital, 1856.—A man aged 56, under the care of Mr. Fergusson, on account of a very osteo-encephaloid growth in the left upper maxilla. It was of twelve years' growth, and he was much reduced in health. An incision, which involved the upper lip only, having been practiced, the cheek was dissected up, and room enough gained to admit of the removal of the entire maxilla. No great amount of blood was lost. Death, with symptoms resembling those of commencing pyæmia, occurred on the fifth day. At the autopsy pus was found in the glands and cellular tissue of the neck.

Case 9.—King's College Hospital, 1856.—A girl aged 16, under Mr. Fergusson's care, for a large osseous growth, involving the right upper maxillary and malar bones. The whole maxilla and part of the malar bone were removed, the only external incision being through the upper lip. The girl recovered well, and left the hospital about a month afterwards.

Case 10.—King's College Hospital, 1856.—A girl, aged 10, under Mr. Fergusson's care. The entire right upper maxilla was excised by the usual operation. The disease proved to be tuberculous deposit in the antrum and surrounding bone. The child, we believe, recovered well.

Case 11.—The Liverpool Royal Infirmary, 1856.—A healthy married woman, aged 23, was admitted under Mr. Bickersteth's care, on account of a solid and hard growth under the left cheek, which caused general enlargement of that side of the face. It was of seven years' growth, and had increased painlessly. In the mouth the left side of the palate was found to be pushed downwards. The case had all the features of an osseous tumor of the antrum and maxilla, and the entire bone was accordingly removed by the usual operation. In extricating the bone profuse hemorrhage suddenly occurred, from the tearing of a large artery (probably the internal maxillary,) but it was arrested by ligature, the carotid having meanwhile been compressed. The patient had an attack of erysipelas afterwards, but she ultimately recovered well. The tumor when sawn across proved to be a bony growth, occupying the interior of the antrum, the whole of which it filled with the exception of a small space capable of holding a pea.

Case 12.—King's College Hospital, 1857.—A robust-looking man of middle age, in whose left upper jaw a tumor had been growing for upwards of four months. The growth extended across the mesial line in the palate, and also involved the malar bone of the opposite side. The whole of the involved maxilla and parts of the malar bones were resected in the usual manner. The man recovered quickly, and was discharged well six weeks afterwards, but Mr. Fergusson expressed himself as having grave fears that the disease would recur.

Case 13.—The Leeds Infirmary, 1857.—A woman, aged 22, was admitted under Mr. Teale's care on account of a large fibrous growth, originally developed within the right upper maxilla, but which in its growth had also involved parts of the left. Excision of the entire right bone was performed, a considerable portion of the left being also removed. The operation involved the loss of much blood, and the patient sank exhausted and died the following day.

Case 14.—The Queen's Hospital, Birmingham, 1857.—A woman, aged 55, under Mr. Langston Parker's care, on account of malignant disease of the right upper maxilla. Excision of almost the entire bone was performed on April 21st. The growth was found to occupy the cavity of the antrum. A good recovery resulted, and at the date of report (four months after) there was no evidence of return of the disease.

Case 15.—The Salford Royal Hospital.—A healthy-looking woman, aged 42, under the care of Mr. Thomas Windsor. The right cheek was distended by a firmly-fixed tumor, which had been growing for four months, and had ulcerated through the palate. On April 15, 1857, Mr. Windsor excised the entire bone, which however, broke into fragments during the removal. There was but little bleeding, and the woman did well for the first twenty-four hours. Subsequently diffuse inflammation of the cellular tissue of the neck followed. From this and from erysipelas of the face she sank on the fourteenth day. The disease was soft cancer, and had begun in the antrum.

Case 16.—St. Thomas' Hospital, July, 1858.—A lad, aged 18, under the care of Mr. Simon, on account of a large polypoid tumor developed in the left upper maxilla. Malignancy was not suspected, but it was evident that the base of the growth could not be got at without extirpation of the bone. The entire maxilla was accordingly resected, and the polypus, which had an exceedingly broad base of attachment to the cranium itself, was got away. The lad recovered well, and, as usual, with but little deformity.

Case 17.—The London Hospital.—In this case, a man, aged 43, under the care of Mr. Adams, had his left upper maxilla excised, on account of a polypoid growth filling the antrum. He made a good recovery.

We have arranged the above seventeen cases in the subjoined table.

It will be seen that out of the seventeen only three ended fatally. In several others there was, however, the disappointing sequel that the disease was malignant, and quickly returned.

TABULAR STATEMENT OF RESECTIONS OF THE UPPER JAW.

| No. | Sex. | Age. | Bone affected. | Nature of Disease. | Result. | Cause of Death. | Remarks. |
|---|---|---|---|---|---|---|---|
| 1 | .. | .. | Left. | Malignant. | Recovered. | .. | The disease rapidly returned. |
| 2 | .. | .. | .. | Fibroid. | Recovered. | .. | Believed to be permanently well. |
| 3 | .. | .. | .. | Malignant. | Recovered. | .. | |
| 4 | M. | 50 | .. | Malignant. | Recovered. | .. | The disease rapidly returned. |
| 5 | F. | 53 | Right. | Malignant. | Recovered. | .. | |
| 6 | M. | 49 | .. | Malignant. | Recovered. | .. | The disease was believed to be returning. |
| 7 | F. | 13 | Left. | Osseous. | Recovered. | .. | Permanent recovery. |
| 8 | M. | 56 | Left. | Osteo encephaloid 12 years. | Died. | Commencing pyæmia fifth day. | The tumor was very large |
| 9 | F. | 16 | Right. | Osseous. | Recovered. | .. | Permanent recovery. |
| 10 | F. | 10 | Right. | Tuberculous | Recovered. | .. | |
| 11 | F. | 23 | Left. | Osseous 7 years. | Recovered. | .. | |
| 12 | M. | 45(?) | Left. | Malignant 4 months. | Recovered. | .. | |
| 13 | F. | 22 | Right. | Fibroid. | Died. | Exhaustion, 1 day. | The tumor was very large |
| 14 | F. | 55 | Right. | Malignant. | Recovered. | .. | |
| 15 | F. | 42 | Right. | Malignant. | Died. | Erysipelas, &c., 2 weeks. | In good health at the time of operation. |
| 16 | M. | 18 | Left. | Polypoid. | Recovered. | .. | The polypus grew by a broad base from the skull itself. |
| 17 | M. | 43 | Left. | Polypoid. | Recovered. | .. | The polypi occupied the antrum. |

OTHER CASES OF MALIGNANT DISEASE OF THE UPPER JAW.

At page 476 of the "Medical Times and Gazette" for November 7, 1857, the reader will find some particulars of a case in which there was little doubt that the upper jaw was involved in a cancerous growth. The patient was a healthy-looking young man of 23, under Mr. McWhinnie's care in St. Bartholomew's Hospital. The performance of excision was declined on account of the growth having advanced too far upwards to permit of it. The right side was affected, and the disease had existed only seven months.

In September, 1854, a man, aged 25, was under the care of Mr. Richard Hey, in the York Hospital, for a malignant tumor occupying the antral cavity. Mr. Hey cut away the front wall of the antrum and scooped out the morbid growths. The parts healed, but we do not know the final result of the case.

An especially interesting case has also been recently under notice, in which a boy from the country, under Mr. Critchett's care at the Ophthalmic Hospital, had cancer of both upper jaws. The disease appeared to have developed itself symmetrically, but from quite distinct points in the two bones. In each instance it grew upwards and displaced the eye. The boy died within a year of its commencement.

RESECTIONS OF PORTIONS OF THE LOWER JAW ON ACCOUNT OF TUMORS.

Case 1.—The Winchester Hospital, 1853.—In this case Mr. Butler excised the right half of the lower jaw on account of a malignant growth from the periosteum. The tumor had been growing for two months, and after removal presented the characters of epithelial cancer. In the course of a month after the operation, and before the wound was quite healed, the disease had returned. Death resulted three months later.

Case 2.—St. Thomas' Hospital, June, 1854.—A man, aged 28, cachectic and of very nervous temperament, was under Mr. Macmurdo's care for malignant disease developed within the bone. It was growing rapidly and expanding the bone on its exterior. The left half of the jaw was excised; the operation being attended with but little loss of blood. The patient did well for about ten days, when he was attacked by erysipelas, under which he sank about a fortnight after the operation. The tumor after removal proved to be of the fibro-plastic or myeloid class. No examination of the viscera after death was permitted.

Case 3.—A Provincial Hospital, 1854.—A woman, aged 40, in very feeble health, had excision of the right half of her lower jaw performed on account of a large fibrous tumor, which had been developed within it, and had been steadily increasing for upwards of three years. Soon after the operation a severe attack of diarrhœa with menorrhagia came on and occasioned much exhaustion. Symptoms of pyæmia subsequently developed themselves, and death followed on the thirty-third day. At the autopsy pus was found beneath the scalp, and there were deposits in the lungs and muscular substance of the heart.

Case 4.—King's College Hospital, April, 1855.—A healthy man, aged 31, was admitted under Mr. Fergusson's care on account of a large tumor in the left side and front part of the lower jaw, which had been slowly increasing for more than three years. The excision involved the removal of the bone from the anterior edge of the left masseter, as far forwards as the canine tooth on the right side. On dissection the bone was found occupied by cysts which contained a gelatinous fluid, its external layer having been expanded until as thin as parchment. The patient recovered well after the operation.

Case 5.—Addenbrooke's Hospital, Cambridge, 1856.—A healthy-looking man, aged 28, was admitted in November, 1856, under Mr. Humphry's care, with a large tumor, resembling an epulis growing from the lower jaw. It was of a year's duration, and involved nearly the entire depth of the bone from the first bicuspid to the second molar. In the operation an incision was carried from the angle of the mouth downwards and backwards nearly to the angle of the bone. The saw was next used, and the tumor, with the whole of the tract of the bone involved, excepting a mere rim at its lower border, was removed. The wound quickly healed, and the man was discharged in about three weeks. Under the microscope cells resembling those of cancer were found.

Case 6.—The Liverpool Royal Infirmary, 1856.—A healthy-looking man, aged 25, was admitted, under the care of Mr. Bickersteth, with a tumor growing from the lower jaw of nine weeks' duration. It had during that period been twice excised by a surgeon under whose care the man had formerly been, but on each occasion a rapid recurrence took place. During the few days he was under

Mr. Bickersteth's observation prior to the operation, it increased in size very rapidly. The horizontal ramus of the jaw was excised to within a short distance of the symphysis. The patient recovered well, but a return of the disease was much feared.

Case 7.—The Liverpool Royal Infirmary, 1856.—A spare man, aged 62, for six months the subject of a firm tumor in the left side of the lower jaw. It was about the size of a walnut, and had been painful. Excision of the portion of jaw from which it grew was performed and the man recovered well. At the time he left the hospital, however, the disease seemed to be returning.

Case 8.—King's College Hospital, 1857.—The subject of this interesting case was a woman, aged 23. When 16 years old, she had first observed a tumor forming in the right side of the lower jaw. This was excised, together with part of the bone, by Mr. Pettigrew. The disease soon returned, and for some time the patient declined to permit any further surgical interference. When admitted, under Mr. Fergusson's care, into King's College Hospital, she still presented the aspect of good health; but the tumor was of large size, involving the angle and ramus of the bone. Mr. Fergusson resected the whole from the first molar tooth to the articulation. The bleeding at the time was very free, and it returned in the course of the evening. Death from collapse followed the next day. The disease proved to be of myeloid structure, and the fact of its recurrence became therefore of especial interest.

Case 9.—The Leeds Infirmary.—In this case the portion of the lower jaw resected was not very large. The patient was a man, aged 33, who had had an epithelial cancer excised from his lower lip about nine months before. Although the cicatrix remained quite sound, he noticed about four months after the operation that the jaw was enlarging. The tumor slowly increased, and became painful. Excision of the affected part of the bone was performed, and with a good result, as the parts healed soundly.

Case 10.—Charing-cross Hospital.—In this instance no distinct tumor existed; but the operation required was the same. The bone was much enlarged and carious at parts, as the result probably of constitutional syphilis. The patient was a sailor, aged 32. Mr. Hancock cut away the left half of the bone from its angle posteriorly to near the symphysis in front. The parts healed well.

Case 11.—The "Great Northern" Hospital, June, 1859. A girl, aged 17, was admitted for the second time under the care of Mr. Lawson. About eight months before a fibrous-looking tumor growing from the periosteum of the left side of the lower maxilla had been removed. The disease had recurred, and now involved the ramus of the jaw in a tumor of considerable size. The ramus of the jaw was exarticulated, and removed, together with the tumor, which grew from its surface. It was found impossible to get away the whole of the growth, as it had spread into the adjacent structures. The girl did well afterwards, but Mr. Lawson had but little hope that the disease would not rapidly recur. It appeared to be of fibroid recurrent character.

TABLE OF RESECTIONS OF PORTIONS OF THE LOWER JAW.

| No. | Sex. | Age. | Bone affected. | Nature of Disease. | Result. | Cause of Death. | Remarks. |
|---|---|---|---|---|---|---|---|
| 1 | M. | .. | Right. | Malignant, 2 months. | Recovered. | .. | The disease returned, and he died in four months. |
| 2 | M. | 28 | Left. | Myeloid. | Died. | Erysipelas, 2 weeks. | The man was in bad health. |
| 3 | F. | 40 | Right. | Fibroid, 3 years. | Died. | Pyæmia, 33d day. | The woman was in very feeble health. |
| 4 | M. | 31 | Left. | Cystic, 3 years. | Recovered. | .. | |
| 5 | M. | 28 | .. | Malignant, 1 year. | Recovered. | .. | |
| 6 | M. | 25 | .. | Malignant, 9 weeks. | Recovered. | .. | |
| 7 | M. | 62 | Left. | Malignant. | Recovered. | .. | A return was feared. |
| 8 | F. | 23 | Right. | Myeloid, 6 years. | Died. | Exhaustion, 1 day. | The tumor was very large. |
| 9 | M. | 33 | Left. | Malignant. | Recovered. | .. | It was secondary on cancer of the lip. |
| 10 | M. | 32 | Left. | Syphilitic enlargement | Recovered. | .. | The bone was enlarged and carious. |
| 11 | F. | 17 | Left. | Fibroid recurrent. | Recovered. | .. | The tumor grew from the periosteum of the ramus. |

### CARTILAGINOUS AND FIBRO-CARTILAGINOUS TUMORS.

Tumors consisting only of cartilage, and resembling those so often developed in the phalanges of the fingers, occur occasionally in connexion with the jaws. They are, however, decidedly infrequent. Mr. Fergusson exhibited to the Pathological Society, in October, 1854, a specimen of this form of tumor, about the size of a hen's egg, which he had removed from the jaw of a woman aged 39. It had existed most of her life, and occupied the interior of the bone between the symphysis and the mental foramen, being encapsuled by a thin osseous shell. Mr. Fergusson remarked that the disease was undoubtedly rare, and that he had never before seen so good a specimen of it. The reporters upon this specimen (Dr. Beale and Mr. Gray) considered it to be more of fibrous than true cartilaginous structure, but Mr. Fergusson describes it "as resembling enchondroma, as seen in the phalanges of the fingers." At a meeting of the Pathological Society during its last session, Mr. Hutchinson showed a fibro-cartilaginous tumor, about the size of a pigeon's egg, which he had excised from the canine fossa of the right upper jaw. The tumor had been movable, occupying a depression in the bone, but not growing from it. The tumor consisted largely of true cartilage. The patient was a girl of about 15, under care at the Metropolitan Free Hospital. Mr. Fergusson, who was president of the society at the time, remarked that such growths in connexion with the jaws were, according to his experience, very unusual. But the best example of cartilaginous tumors growing in the jaws with which we have recently been made acquainted, is one, the particulars of which Dr. Adams, of the Richmond Hospital, Dublin, recorded in the "Medical Times and Gazette" for April 11, 1857. In it "the whole body of the lower jaw was expanded into a bony cyst, or shell, which enclosed within it a cartilaginous-like material."

The excision required involved the removal of the articular head as well as the body and ramus. The patient was 36 years of age, and the disease had followed a blow received three years before. These tumors are of course perfectly innocent. They usually commence in early life and grow very slowly.

### EPULIS.

In the following list of examples of epulis which have been operated on in our various hospitals, and recorded from time to time in our statistical reports, we have included several in which there was much doubt as to whether the tumor was not really malignant. But in truth, in respect to these tumors of the gum and alveolus, it is often very difficult to estimate their degree of deviation from the benign standard. The fibrous epulis is certainly not malignant, but it often returns after free excision. The myeloid epulis stands on more questionable ground, will ulcerate and fungate, and now and then may cause enlargement of glands, whilst a third class presents itself which is more suspicious, whilst yet not by any means possessing clear and positive characteristics of cancer. Our data have not enabled us in the following list to distinguish between fibrous and myeloid, but we have mentioned the circumstance whenever the disease was believed to be malignant.

TABULAR STATEMENT OF TWENTY-EIGHT CASES OF EPULIS.

| No. | Sex. | Age. | Duration. | Position. | Result. | Remarks. |
|---|---|---|---|---|---|---|
| 1 | F. | 35 | .. | Upper. | Recovered. | |
| 2 | M. | 39 | 15 months. | .. | Recovered. | |
| 3 | F. | 60 | 7 months. | Upper. | Recovered. | |
| 4 | F. | 50 | 3 months. | Upper. | Died. | Rigors followed the operation, and death from pyæmia on the 15th day. |
| 5 | M. | 16 | 3 years. | Lower. | Recovered. | |
| 6 | F. | 60 | 20 years. | Upper. | Recovered. | Very large indeed. It had returned after removal 8 years before. |
| 7 | F. | 26 | 9 months. | Lower. | Recovered. | Large, ragged, and fungating. It was fibro-cartilaginous. |
| 8 | M. | 36 | .. | Upper. | Recovered. | It was thought after removal to be of cancerous nature. |
| 9 | M. | 27 | 7 years. | Upper. | Recovered. | The tumor was thought to be cancerous after removal. |
| 10 | F. | 28 | 6 years. | Lower. | Recovered. | The tumor consisted of hardish bone, and had encapsuled completely the stumps of two teeth. |
| 11 | F. | 11 | .. | Lower. | Recovered. | |
| 12 | F. | 36 | 18 months. | Lower. | Recovered. | Caused by a decayed tooth. |
| 13 | M. | 24 | .. | .. | Recovered. | |
| 14 | F. | 30 | .. | Upper. | Recovered. | |
| 15 | F. | 23 | 14 months. | Lower. | Recovered. | Two bicuspid teeth were buried in it. It was of myeloid structure. |
| 16 | F. | 22 | 2 years. | .. | Recovered. | It involved two teeth. |
| 17 | M. | 16 | 1 year. | Lower. | Recovered. | It involved the last bicuspid and first molar. |
| 18 | F. | 31 | .. | Lower. | Recovered | The tumor was soft and fungoid. |
| 19 | F. | 30 | .. | Lower. | Recovered. | |
| 20 | M. | 9 | .. | .. | Recovered. | |
| 21 | F. | 22 | .. | Lower. | Recovered. | |
| 22 | M. | 40 | .. | .. | Recovered. | |
| 23 | M. | 40 | .. | .. | Recovered. | It was ulcerated, and considered to be malignant. |
| 24 | M. | 10 | .. | Lower. | Recovered. | As large as a walnut. |
| 25 | M. | 51 | .. | Upper. | Recovered. | |
| 26 | F. | 47 | 5 months. | Upper. | Recovered. | |
| 27 | F. | 24 | .. | Lower. | Recovered. | The bleeding which followed required the actual cautery for its arrest. |
| 28 | F. | 73 | .. | .. | Recovered. | The tumor was pedunculated, and was removed by ligature. |

Of these 28 cases, in which tumors growing from the gum were of the character usually designated as "epulis," we may make the following summary:—In but one instance did the operation cause the death of the patient, whilst in all the others the parts implicated are stated to have healed soundly. It would appear that the female sex is more liable to this disease than males, in the proportion of 5 to 3, the numbers in the list being 17 females and 11 males. This may perhaps be explained by reference to the fact, that stumps of decayed teeth are by far the most frequent exciting causes of these growths. Now women are, for several reasons, more likely to retain useless stumps of teeth than men. They are far more patient as regards severe, unavoidable pain, such as that of toothache, and at the same time much more afraid of surgical pain, as that of tooth extraction ; besides, it must be remembered, that the conditions either of pregnancy or lactation prevent many women from having their decayed teeth taken out at the time when they ache.

As it regards *age*, we find that the youngest patient was a boy of 9, and the next to him a girl of 11, whilst the oldest was a woman of 73, and the next to her another woman of 60. Five were under the age of 20 ; eight between those of 20 and 30 ; seven between 30 and 40 ; three between 40 and 50 ; two between 50 and 60 ; and three above 60. The average of the whole is 33.

The lower jaw seems rather the more frequent seat of epuloid tumors. In twelve out of twenty-one cases in which we possess information on this point, the growth was from the lower gum, and in nine only from the upper.

It is a curious fact, that epulis tumors are hardly ever seen in the front gum, but almost invariably at one or the other side. Of course the greater extent of the lateral regions, and the fact that for each central tract there are two lateral ones, explain this to some extent. The increased irritation of the many-fanged molars, as compared with the incisors, must be taken also into the account.

### MULTILOCULAR CYSTIC DISEASE.

In case 4 of the above series of excisions of parts of the lower jaw, the disease is stated to have consisted of numerous cysts filled with gelatinous material, which occupied the body of the bone. The osseous shell was, in many parts, as thin as parchment. A parallel case to this has also been recorded by the Dublin surgeon just alluded to (Dr. Adams.) The patient was a man, aged 36, and the disease had existed for three years. As in Mr. Fergusson's case the cysts were developed in the body of the lower jaw, and almost a similar operatiou was required. In the same report Dr.

Adams refers to (and figures the specimen) a third case of cystic disease of the lower jaw, which had been recorded by Mr. Cusack many years before.

FIRM FIBROUS TUMORS.

In January, 1858, Mr. Cock had under his care, in Guy's Hospital, a boy, aged 9, in whom a tumor was embedded in the left side of the lower jaw, near to its symphysis. The growth had followed a blow received two years before. It was excised, and proved to be a firm fibrous tumor, of about the size of a walnut. It was not encapsuled in the bone at the time of the operation, but occupied a depression in its surface. It surrounded the mental nerve, and had probably been originally developed in its canal. Respecting several of the cases in the above series in which the term "fibroid" has been used, we are at some loss how to class them. Some so described were possibly truly fibrous; others probably of myeloid nature; and others, again, very closely allied to cancer, or, at any rate, to the recurrent fibroids. The above is the only undoubted instance of an ordinary fibrous tumor developed in connection with either jaw, that has recently come under our notice.

*Exostosis from the Lower Jaw.*

In one instance Mr. Erichsen removed from near the symphysis of the lower jaw an exostosis of moderate size, which had been increasing several years. The patient was a woman, aged 22. This is, we believe, the only example of exostosis developed in this situation which our lists contain.

*Recurrent Fibroid Tumor Developed in the Antrum.*

The only example which we have to quote of recurrent fibroid tumor developed in connexion with the jaws is one in which the diagnosis of that variety of tumor and true cancer is by no means positive. It is that of a woman, aged 34, under Mr. Cock's care, in Guy's Hospital, at different times for two or three years (1854 and 1856.) The growth occupied the right antrum, and extended into the nose; on several occasions Mr. Cock dissected up the cheek in front, laid bare the cavity, and gouged out the tumor and the bone to which it was attached. The parts always healed quickly, but the disease soon returned. The tumor had the microscopic features of a recurrent fibroid, as distinct from those of a true cancer, and the fact that it continued to recur in the same place, but did not cause disease of the glands, is confirmatory of that diagnosis. The woman was very pallid and cachectic, but her cachexia did not exactly resemble that of cancer. We lost sight of her towards the end of 1856, and do not know the final result of her case. Probably she has since died of her disease.

*Cystic Distension of the Antrum.*

Several good examples of cystic distension of the antrum have been recorded in the Medical Times and Gazette during the period now under review. In this curious affection the antral cavity becomes filled with a glairy mucous fluid closely resembling that of ranula, the outer wall is distended and thinned, and the cheek becomes visibly full and swollen. Often on touching the cheek, a sensation of crackling as of paper is produced, owing to the extreme thinness of the external osseous wall. On opening the antrum it is found of great capacity, but not containing any morbid growths, either solid or membranous. The disease is, therefore, rather a distension of a natural cavity than a true cystic formation. It would appear that women are more subject to this affection than men.

The following are examples of it:—Case 1.—A woman, aged 31, was admitted in October, 1855, into Guy's Hospital, under the care of Mr. Cock. Her right antrum was so distended that the osseous wall in front had been wholly absorbed. Mr. Cock dissected upwards between the cheek and gum, and cut away with strong scissors as much as possible of the front wall of the cyst, which was tough and fibrous. The glairy contents having been evacuated, the cavity was stuffed with sponge and left to suppurate. When the woman left the hospital the cyst appeared to have been obliterated.

Case 2.—Under the care of Mr. Teale, in the Leeds Infirmary. It was necessary to employ bone forceps in cutting away the front wall of the dilated antrum.

Case 3.—A girl, aged 14, under the care of Mr. Hilton, in Guy's Hospital, on account of a large prominent swelling of the left upper maxilla. Mr. Hilton diagnosed a cyst either in or connected with the antrum. The operation consisted in dissecting up the gum and soft parts, and then with a strong pair of scissors opening the front wall of the swelling, which consisted of thin and expanded bone. Nearly two ounces of thick, glairy fluid escaped. Some lint was afterwards introduced into the wound. After some slight suppuration, during which a lotion of the sulphate of zinc was used as an injection, the cavity filled by granulation, and the wound closed. The cure was complete, and all deformity of the cheek subsequently disappeared.

Case 4.—The patient was a woman of about 35, under Mr. Hutchinson's care in the Metropolitan Free Hospital. The cyst had been opened several times, but always when the puncture closed it refilled. On one occasion, at another hospital, an attempt had been made to prevent the opening from healing, but unsuccessfully. In front was a thin shell of bone. Mr. Hutchinson laid the cyst freely open, and stuffed it with sponge, which was left in for four days. After the removal of the sponge, injections of chlorate of potash were employed to correct the fetor of the discharge. It is believed that the obliteration was complete.

### *Cyst in the Upper Jaw, Enclosing a Tooth.*

Of this peculiar class of cases we have but one example, which is as follows :—

A young woman, aged 18, admitted into Guy's Hospital under the care of Mr. Cock, having for some time suffered from a painful swelling of the gum of the left side of the upper jaw, over the incisor teeth. An incision had been made into it by a surgeon who had previously seen her, and a portion of gum removed, but without benefit. It was found that the permanent lateral incisor tooth of that side had not appeared, and that its position was still occupied by a small deciduous one. Mr. Cock's diagnosis was that the tumor was probably connected with some malposition of the permanent tooth. Mr. Salter, the dentist to the hospital, saw the patient, and extracted the deciduous tooth, which was found firmly fixed, and its fang unabsorbed. No further information as to the nature of the swelling in the jaw was gained by this measure. Mr. Cock determined, accordingly, to open the swelling and examine. This was done by means of small bone forceps, by which, after the gum had been detached, a portion of the anterior part of the jaw, just above the alveolar process, was cut away. A cavity of irregular shape, and about capable of containing a large marble, was laid open, and after some search the wanting tooth was found at its further extremity. The tooth was a large, fully-formed, permanent incisor, beautifully white, and complete in every respect, excepting that its fang terminated abruptly in a rounded end at about its middle. The wound was left to heal by granulations, which it rapidly did without any inconvenience. It is intended, by the aid of the dentist, to have the tooth fixed in the position it should have occupied.

